# Revisiting phage tail spike architecture: evidence for undetected receptor-binding proteins in *Caudoviricetes* with non-contractile tails

**DOI:** 10.3389/fmicb.2025.1625765

**Published:** 2025-07-16

**Authors:** Rafał Matusiak, Magdalena Antczak, Anna Kawa, Małgorzata Paszkiewicz, Jolanta Witaszewska, Joanna Kazimierczak, Ewelina A. Wójcik

**Affiliations:** Research and Development, Proteon Pharmaceuticals S.A., Łódź, Poland

**Keywords:** bacteriophages, receptor recognition mechanisms, tail spike, receptor binding protein, structural bioinformatics

## Abstract

**Introduction:**

Bacteriophages, viruses that infect bacterial cells, have garnered renewed interest as potential therapeutic agents due to the growing threat of antibiotic resistance. Effective application of bacteriophages requires a comprehensive understanding of their structure and mechanisms of action. Recent advances in structural biology techniques, such as cryo-electron microscopy (Cryo-EM) and cryo-electron tomography (Cryo-ET), along with significant progress in genome sequencing and bioinformatics, have greatly enhanced our knowledge of bacteriophage biology. However, these techniques remain insufficient in some cases to fully resolve the structure and function of phage tail spikes and tail fibers.

**Methods:**

This study investigates the receptor-binding proteins (RBPs) of bacteriophages within the *Caudoviricetes* family, which recognise various receptors on bacterial surfaces. Bioinformatic analysis involving protein complex modelling with AlphaFold2-Multimer and molecular dynamics simulations was employed to reveal the evolutionary conservation and structural diversity of RBPs across different phage genera.

**Results:**

Our findings indicate that phages from the genera *Dhillonvirus*, *Traversvirus*, and *Littlefixvirus* lack a receptor-binding domain at the distal end of the central tail spike. Furthermore, we identified and reconstructed previously unannotated or misannotated proteins that may contribute to receptor recognition.

**Discussion:**

These results suggest that the analysed phages possess an additional, previously unidentified protein at the tip of the tail spike, which likely facilitates interaction with receptor proteins on the bacterial cell surface.

## Introduction

With the rise of antibiotic resistance, phage therapy is experiencing a resurgence. Recent studies have shown that bacteriophages—not only through the direct lysis of bacterial cells but also by modulating bacterial virulence and enhancing antibiotic sensitivity via directed evolution—hold promise as innovative agents in sustainable medicine (Kebriaei et al., 2023; Chan et al., 2016; Gurney et al., 2020).

After decades of relative scientific dormancy, interest in bacterial viruses has been revitalized by advances in structural biology, particularly cryo-electron microscopy (Cryo-EM) and cryo-electron tomography (Cryo-ET), which have enabled detailed analyses of macromolecular structures (Li et al., 2023; Arnaud et al., 2017; Fokine et al., 2007; Luque and Castón, 2020; Šiborová et al., 2022). The explosive growth in sequencing technologies and bioinformatics tools has also helped to resolve many longstanding questions in phage biology. In recent years, these advances—further enhanced by deep learning algorithms—have enabled the development of powerful new methods for virion structure prediction and protein reconstruction (Cantu et al., 2020; Gligorijević et al., 2021; Shor and Schneidman-Duhovny, 2024). Today, structural analysis can be carried out even without high-resolution data, allowing for a deeper understanding of virion mechanics and the simulation of infection processes.

Focusing on the Caudoviricetes class, it is important to note that these phages typically recognize at least one receptor on the bacterial cell surface via receptor-binding proteins (RBPs). Initial attachment involves interactions with first or supporting receptors, or the enzymatic degradation of capsular polysaccharides that otherwise hinder access to the main or second receptor. In many cases, final irreversible binding triggers conformational rearrangements of the phage tail and the formation of an anchoring structure through exposure of hydrophobic regions to the host membrane (Steven et al., 1988; Cuervo et al., 2013; Garcia-Doval et al., 2015; Taylor et al., 2016; Hyman and van Raaij, 2018; Linares et al., 2023). *Caudoviricetes* virions display a remarkable diversity of tail structures. For instance, *Demerecviridae* phages feature non-contractile tails and three tail fibers (pb1) that recognize supporting receptors, often carbohydrate motifs. The main receptor is recognized by pb5, which is attached to a homo-trimer of pb4 and forms a flexible tail in conjunction with pb3 (Garcia-Doval et al., 2015; Linares et al., 2023). In contrast, *Straboviridae* phages possess contractile tails with two sets of six fibers: long fibers ending in gp37 or gp38 for receptor recognition (either sugar motifs or outer membrane proteins), and short fibers (gp12) that irreversibly bind to the LPS core Additionally, they have short stiff central tail spikes with three lysine domains created by a trimer of gp5 (Yap et al., 2016; Hyman and van Raaij, 2018). *Autographiviridae* phages have short, non-contractile tails and mostly six fibers; here, the main receptor is recognized by a homo-trimeric tail fiber, followed by interaction of a homo-hexameric tail spike with the LPS core and attachment to cell membrane (Steven et al., 1988; Cuervo et al., 2013; Huss et al., 2021; Swanson et al., 2021).

Although *Caudoviricetes* were analysed for over half a century, the mechanisms underlying their recognition and binding to outer membrane porins remained elusive for decades. Recent structural studies on *Tequintavirus T5* (Linares et al., 2023) and *Lambdavirus lambda* (Wang et al., 2024)—both of which have long, non-contractile tails and use outer membrane proteins as receptors—revealed architectural similarities in their central tail spike recognition systems. In *Tequintavirus T5*, the additional protein pb5, which forms an immunoglobulin-like structure, is attached to the C-terminal ends of a pb4 homo-trimer and is responsible for receptor recognition. In *Lambdavirus*, the tail spike protein gpJ contains immunoglobulin-like domains at its C-terminus, which form three receptor-binding epitopes displayed at the tip.

The structural and functional analysis of the phage tail prepared by Chang Wang and Romain Linares in their works show that the most important elements of the phage receptor recognition system are immunoglobulin-like domains exposed at the end of homo-trimer tail spike protein or additional protein attached to conservative tail spike trimer (Linares et al., 2023; Wang et al., 2024). The bioinformatic analysis of bacteriophages from different families that also recognise outer membrane proteins shows that members of the *Dhillonvirus* and *Traversvirus* genera possess tail spikes structurally similar to gpJ of *Lambda*, but notably lack immunoglobulin-like domains at the C-terminal end Lewis and colleagues have demonstrated that *Dhillonvirus JLBYU60* uses the FhuA protein as its main receptor and is sensitive to deletions in loops L3, L5, and L8 (Lewis et al., 2023). Similarly, Smith’s research on *Traversvirus tv24B* revealed resistance phenotypes in *bamA* deletion mutants (Smith et al., 2007).

The absence of Ig-like domains in the tail spike receptor-binding proteins of *Dhillonvirus JLBYU60*, *Traversvirus tv24B*, and other phages—such as *Littlefixvirus 98PfluR60PP* (from the Proteon Pharmaceuticals collection)—suggests the involvement of alternative structural elements. In this study, we conduct a comprehensive bioinformatic investigation of receptor-binding systems that recognize outer membrane porins as primary receptors in phages from the *Dhillonvirus*, *Traversvirus*, and *Littlefixvirus* genera. Our analysis identifies multiple poorly annotated or misannotated proteins that may play key roles in receptor recognition. Notably, we present the first in silico reconstruction of these systems, highlighting functional elements that may have been overlooked by conventional annotation methods.

## Materials and methods

### Bioinformatic analysis

The genome and proteomes sequences of the analyzed phages were obtained from the NCBI Reference Sequence database: *Tequintavirus T5* (GenBank, accession no. NC_005859.1), *Lambdavirus lambda* (GenBank, accession no. NC_001416.1), *Dhillonvirus JLBYU60* (GenBank, accession no. NC_073052.1), *Traversvirus tv24B* (GenBank, accession no. NC_027984.1), *Littlefixvirus 98PfluR60PP* (GenBank, accession no. NC_070866.1).

Identity matrices for the analyzed proteins were generated using Clustal v2.1 (Madeira et al., 2019). Protein homology and sequence identity were assessed with BLASTp v2.9.0 (Camacho et al., 2009). Functional annotation and identification of conserved domains were performed using the NCBI Conserved Domain Database (accessed 2023–2024, https://www.ncbi.nlm.nih.gov/Structure/cdd/wrpsb.cgi) (Wang et al., 2023) and InterProScan v5.62–94.0 (Jones et al., 2014). Distant homologs of the analyzed proteins were identified using the Foldseek server (accessed 2023–2024, https://search.foldseek.com/) against comprehensive structural databases (van Kempen et al., 2024).

Protein homology modeling was conducted via the SWISS-MODEL server^1^ (Waterhouse et al., 2018). The new receptor-binding proteins (RBPs) and protein oligomers were modeled using AlphaFold2 v2.3.2 (Jumper et al., 2021) and AlphaFold2-Multimer v3 (Evans et al., 2021), and implemented through ColabFold v1.5.2 (Evans et al., 2021; Jumper et al., 2021; Mirdita et al., 2022). The modeling protocol included template-based modeling with 30 recycles, and structure relaxation was performed using the AMBER (Assisted Model Building with Energy Refinement) option. The affinity of predicted protein structures to the planar outer membrane of Gram-negative bacteria was evaluated using the PPM 3.0 Web Server^2^ (Lomize et al., 2022).

The *Lambdavirus lambda* protein NP_040600.1 was reconstructed by combining the N-terminal model—generated by SWISS-MODEL in its native homo-trimeric state—with a C-terminal model from AlphaFold2-Multimer v3. Assembly and refinement were carried out in ChimeraX v1.7 (Pettersen et al., 2021). Tail spike tip regions were digitally assembled using AlphaFold2-Multimer v3, restricting the input sequence to the final 200 amino acids of the C-terminus. Oligomeric states were inferred based on structural similarity to viral proteins with experimentally validated oligomeric conformations. The same procedure was applied to protein YP_010740310.1 from *Dhillonvirus JLBYU60*.

Protein-receptor interactions were predicted using heteromeric modeling in AlphaFold2-Multimer v3 with templates and 30 recycles. All multimeric predictions were manually curated to exclude biologically implausible interactions, particularly with membrane-embedded receptor regions inaccessible to RBPs. Modeling focused on phages with experimentally validated receptors: *Dhillonvirus JLBYU60* and *Traversvirus tv24B*. For FhuA (UniProtKB ID: P06971), the full protein sequence was used, whereas for BamA (UniProtKB ID: P0A940), only the C-terminal β-barrel transmembrane domain (residues 427–810) was modelled.

The functional annotations were performed by Sphae snakemake workflow performed by Bhavya Papudeshi and colleagues (Papudeshi et al., 2025). This workflow allows made functional annotations by Pharokka (Bouras et al., 2023), Phold, which functional annotation using protein structures for determining the structural homology (unpublished), repository available^3^ and Phynteny, which is the synteny-based annotation of bacteriophage genes (Grigson et al., 2023). The protein sequences were also analysed by PHROG Sequence search for detection matching the Prokaryotic Virus Remote Homologous Groups (Terzian et al., 2021).

Predicted binding affinities of protein complexes were evaluated using the PRODIGY Web Server^4^ (Xue et al., 2016). Molecular dynamics (MD) simulations were conducted using GROMACS v2023.3 (Páll et al., 2020) with the CHARMM27 all-atom force field (Brooks et al., 1983, 2009). Simulations ran for 1 μs in a 0.15 M NaCl aqueous environment at 300 K. MD trajectories were analyzed using the MDAnalysis Python library v2.7.0 (Michaud-Agrawal et al., 2011), and root-mean-square deviation (RMSD) plots were generated with Plotly (Plotly Technologies Inc., Montréal, QC, 2015, https://plot.ly). Protein structures and cryo-EM maps were visualized and analyzed in ChimeraX v1.7, while MD trajectories were further examined using VMD v1.9 (Humphrey et al., 1996). Visualization videos were rendered using Adobe Premiere Elements 15 (Adobe Inc., San Jose, USA).

### Phage propagation and purification

The bacteriophage *Littlefixvirus 98PfluR60PP* was propagated in a host bacterial culture. The culture medium was inoculated with the host strain and incubated at 25°C for approximately 3 h. Subsequently, the phage was added to the culture, followed by an additional 4-h incubation at 25°C to allow for host cell lysis. This procedure yielded a high-titer phage preparation, reaching approximately 1 × 10^9^ PFU/mL.

Following amplification, unlysed cells and cellular debris were removed by filtration. The resulting phage-containing supernatant was further purified and concentrated by cesium chloride (CsCl) ultracentrifugation at a final CsCl concentration of 1.5 g/mL. To prepare the gradient, 13.2 mL of phage suspension was gradually mixed with 9.9 g of CsCl (Thermo Scientific, J65950.22) and divided into three 4.4 mL ultracentrifuge tubes. Ultracentrifugation was performed for 23 h at 38,000 rpm (150,000 × g) at 4°C using a Thermo Scientific Sorvall™ WX + ultracentrifuge with the swinging bucket rotor (Thermo Scientific TH-660 rotor). The visible white-to-grey band containing phages was collected using a sterile needle.

To remove residual CsCl, the sample was dialyzed first for 2 h, and then overnight at 8°C to SM buffer (100 mM calcium chloride, 8 mM magnesium sulfate, 50 mM Tris–HCl, gelatine 0.01%), using Slide-A-Lyzer MINI Dialysis Devices (10 K MWCO; Thermo Scientific). The final purified phage preparation was subjected to titer determination via a double agar overlay plaque assay.

### SDS-PAGE of virion protein

Purified phage particles were subjected to one-dimensional sodium dodecyl sulfate–polyacrylamide gel electrophoresis (1D SDS-PAGE) following the protocol described by Boulanger (2009). Phage protein samples were heat-denatured and subsequently separated on a 12% polyacrylamide gel (Bio-Rad). The gel was then stained using Instant Blue (Expedeon) to visualize protein bands.

## Results

### The bioinformatic analysis of the evolutionary conservation of the receptor binding system among bacteriophages from the *Caudoviricetes* family

**Figure 1:**
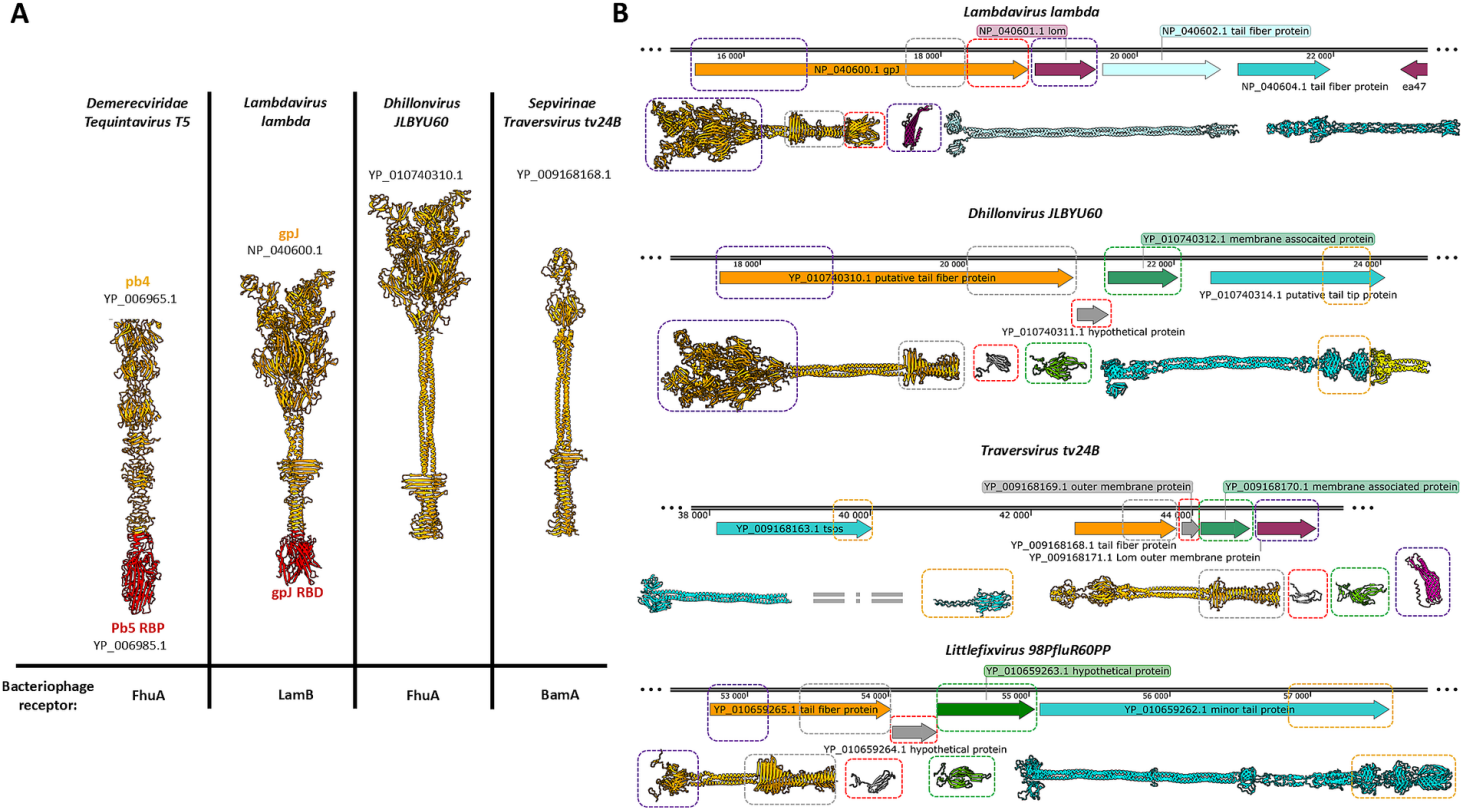
The comparison of the models bacteriophage’s tail spike receptor binding proteins from different families, and the comparison of the regions encoding the receptor binding systems formed by a central tail spike and tail fibers. **(A)** The receptor binding proteins or receptor binding domains are presented in red. **(B)** The figure shows parts of the genomes of four different bacteriophages belonging to the evolutionary distant taxonomical groups. The open reading frames encoding the tail spike are orange. The regions encoding tail fibres are cyan. The open reading frames encoding small proteins with immunoglobulin domain are grey. The regions encoding a structural conservative protein with unknown functions are green. The protein YP_010740314.1 consists of an intramolecular autocatalytic chaperon that is yellow. The yellow rectangular marks the carbohydrate-binding domains. The violet rectangular marks detected tail spike’s N-terminal domain. The grey rectangular marks detected tail spike’s knife-like C-terminal domain. The red rectangular marks detected immunoglobulin-like domain. The green rectangular marks detected unknown hypothetical structural conservative protein. The magenta rectangular marks Lom and Lom-like protein.

A comparative analysis of the genomic regions encoding receptor-binding systems and their corresponding protein models revealed notable similarities among *Dhillonvirus JLBYU60*, *Traversvirus tv24B*, and *Littlefixvirus 98PfluR60PP*, as well as a correlation with *Lambdavirus lambda*, despite the considerable evolutionary distance between these phages (Figure 1A). Several structurally conserved domains and proteins identified in the aforementioned genomes are highlighted in Figure 1B (indicated by colored rectangles).

*Lambdavirus lambda* is a non-enveloped bacteriophage characterized by a long, non-contractile tail and extended lateral tail fibers. Its receptor-binding system comprises three proteins. The first, gpJ (NP_040600.1), forms the central tail spike and contains an immunoglobulin-like (Ig-like) domain at its C-terminus, which recognizes the primary receptor, the LamB protein. The second (NP_040602.1) and third (NP_040604.1) proteins constitute the tail fibers. The homo-trimer of NP_040604.1 forms the distal portion of the tail fiber and functions as a supporting receptor-binding protein. Structurally, this homo-trimer is similar to the C-terminal domain of gp37 from phage T4, which interacts with the OmpC porin. In lambda phage this region is responsible for binding both outer membrane porins C and F (Meyer et al., 2012). Between the genomic regions encoding the tail spike and the tail fibers lies an ORF encoding the outer membrane protein Lom (NP_040601.1), a β-barrel protein with an N-terminal membrane localization signal peptide. Lom is incorporated into the host membrane during infection and is expressed during lysogeny in *E. coli*.

*Dhillonvirus JLBYU60*, another non-enveloped bacteriophage with a long non-contractile tail and long lateral fibers, has been shown by Lewis et al. (2023) to use the ferrichrome outer membrane transporter (FhuA) as its main receptor. Bioinformatic analysis revealed a receptor-binding system organization comparable to that of *lambda* (Figure 1B). The system includes both tail fibers and a tail spike. The terminal regions of the tail fiber proteins possess carbohydrate-binding domains and intramolecular autocatalytic chaperones, suggesting an affinity for sugar motifs. Structural modelling indicated that the tail spike lacks a C-terminal Ig-like domain, instead terminating in a knife-like fold. The ORF immediately downstream encodes a small hypothetical protein (YP_010740311.1) with a β-sheet-rich structure, confirmed through structural modelling. The following ORF encodes a 23 kDa protein (YP_010740312.1), annotated by Lewis et al. as a putative membrane-associated protein. Modelling of this protein revealed a novel hand-like β-sheet fold (Figure 1B).

**Figure 2:**
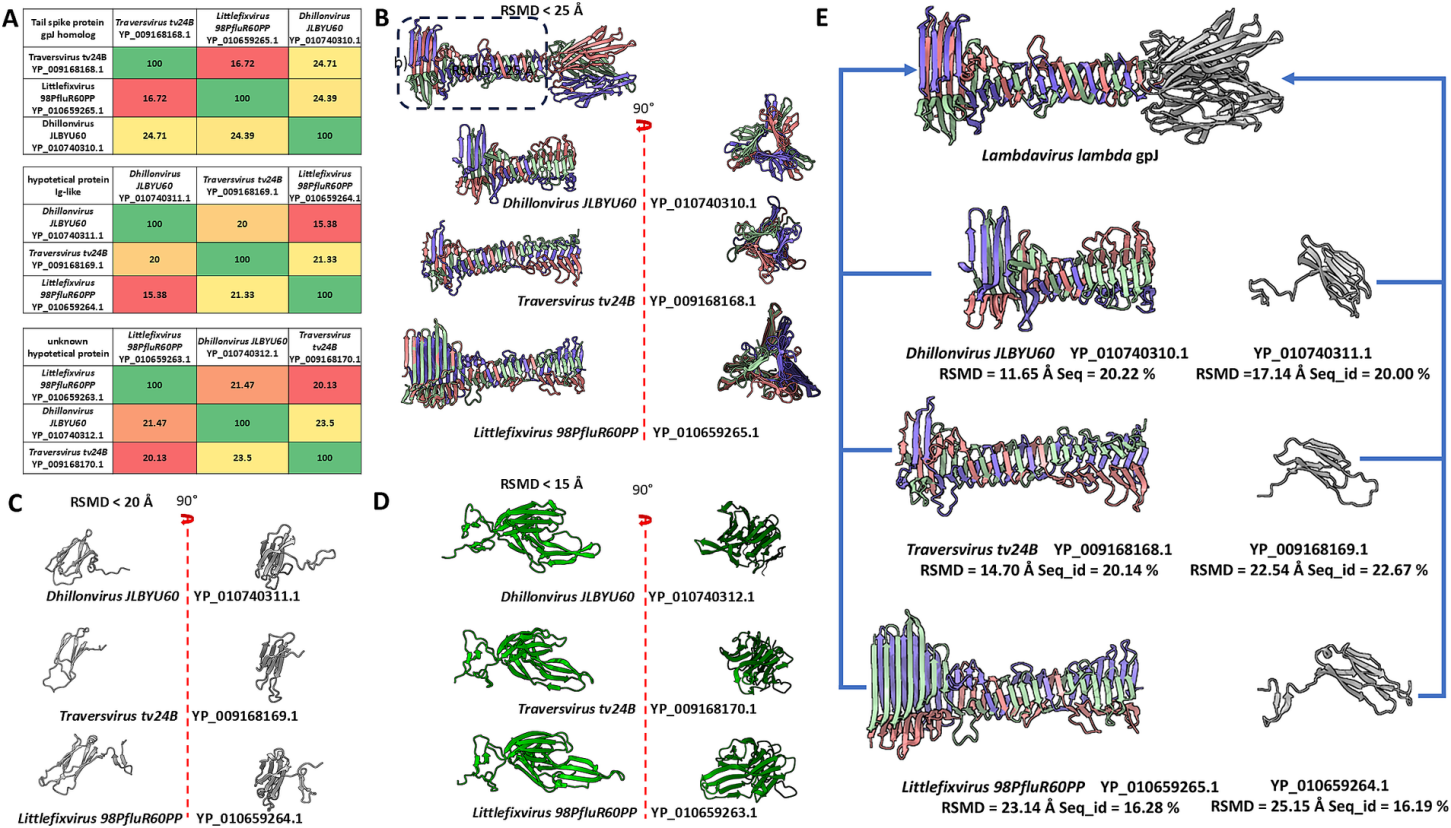
The comparison of sequences and models of analysed domains and proteins, and the comparison of tail spike C-terminal domains of analysed phages and Immunoglobulin-like protein structures to the structure of receptor binding domain of *Lambda* phage (NP_040600.1 gpJ). **(A)** The matrix of sequence identity of compared elements. **(B)** The comparison of the models of the tail spike C-terminal domains “the knife-like domain” in analysed phages. **(C)** The model comparison of the Ig-like small proteins encoded by orf after orfs encoding tail spike. **(D)** The model comparison of unknown proteins mainly annotated by the sequence authors as “membrane associated protein.” **(E)** The predicted model were compared to the some part of the *Lambdavirus lambda* gpJ C-terminal domain. The blue arrows presents which model were compare with which element of the gpJ C-terminal domain.

The next two analyzed phages, *Traversvirus tv24B* and *Littlefixvirus 98PfluR60PP*, are small non-enveloped phages with short, non-contractile tails and long lateral fibers. In *Traversvirus tv24B*, genome annotation showed an ORF encoding a supporting receptor-binding protein (YP_009168163.1) located ~2000 nucleotides upstream of the tail spike-encoding region. Structural analysis revealed that this protein consists of a C-terminal sugar-binding domain. The subsequent protein (YP_009168168.1) encodes a typical tail spike structurally similar to *lambda*’s gpJ, including N-terminal domains required for virion attachment, but lacking a C-terminal Ig-like domain—paralleling the configuration seen in *Dhillonvirus JLBYU60*. Structural modelling of the downstream ORF product (YP_009168169.1), annotated as an outer membrane protein, showed high structural similarity to YP_010740311.1 from *Dhillonvirus*. Likewise, the next downstream protein (YP_009168170.1), annotated as a membrane-associated protein, was structurally similar to YP_010740312.1 from *Dhillonvirus* (Figure 2). This protein, which occupies the third position in the receptor-binding region, was also annotated by the original submitters as a Lom-like outer membrane protein—an annotation confirmed by our structural bioinformatics analysis (Figure 1B).

In *Littlefixvirus 98PfluR60PP*, the genomic arrangement of ORFs encoding the receptor-binding system mirrors that of *Dhillonvirus JLBYU60*. Structural modelling showed that the tail spike protein is architecturally similar to those described above. No Ig-like receptor-binding domain was detected. Instead, downstream ORFs encode small proteins that are structurally similar to those found in *Dhillonvirus* and *Traversvirus*, although annotated here as hypothetical proteins. The final protein in the receptor-binding module (YP_010659262.1) is annotated as a minor tail protein. Structural modelling of this protein in its predicted homo-trimeric state indicates that it forms tail fibers with C-terminal carbohydrate-binding domains (Figure 1B).

Sequence and structure comparisons of these proteins are presented in Figure 2A. Despite low sequence identity, structural modelling revealed significant conservation among proteins encoded downstream of the tail spike genes.

Further comparison between the C-terminal domains of tail spikes and small Ig-like proteins encoded downstream of the spike-encoding ORFs revealed strong structural similarity to the C-terminal Ig-like domain of *lambda*’s gpJ (NP_040600.1), although in the analysed phages, these domains appear to be encoded as separate ORFs (Figure 2E).

Proteins encoded downstream of the Ig-like domain ORFs were generally annotated as membrane-associated proteins (represented in green in Figures 1, 2). Membrane affinity estimation (Table 1) showed that these hypothetical proteins exhibit significantly lower predicted affinity for the bacterial membrane compared to *lambda*’s Lom protein, suggesting that they likely do not embed into the membrane. Based on genomic localization and transcriptional direction, we hypothesize that these proteins are expressed between the tail spike and tail fiber genes and may serve structural or chaperone-like roles in tail assembly.

**Table 1:** Comparison of the affinity of Lom protein and conservative hypothetical proteins encoded by analysed phages to the outer cell membrane of gram-negative bacteria.

Protein ID	Protein name	Depth/Hydrophobic Thickness	ΔG transfer [kcal/mol]	Tilt Angle	Model
NP_040601.1	*Lambdavirus lambda* Lom	24.9 ± 1.3 Å	−43.8	7 ± 3°	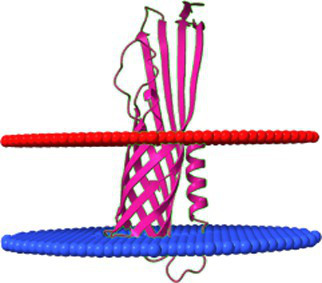
YP_009168170.1	*Traversvirus tv24B* membrane associated protein	3.6 ± 2.0 Å	−6.1	57 ± 3°	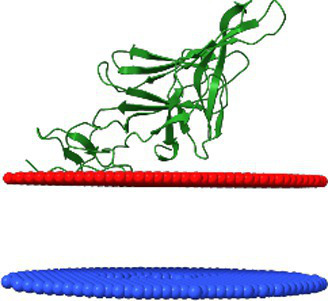
YP_010659263.1	*Littlefixvirus 98PfluR60PP* hypothetical protein	2.5 ± 2.0 Å	−4.9	84 ± 2°	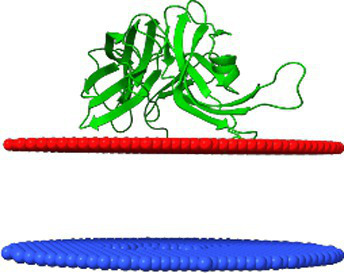
YP_010740312.1	*Dhillonvirus JLBYU60* membrane associated protein	4.5 ± 1.5 Å	−3.8	17 ± 12°	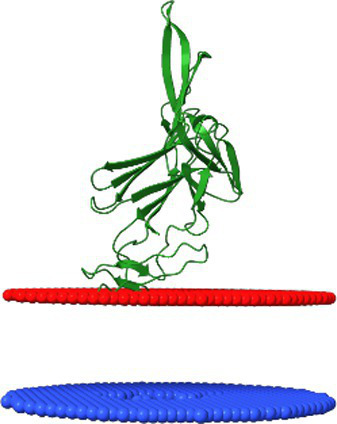

For the assessment of the possibility of analysed proteins interacting with the cell membrane, the maximum penetration depth of protein atoms into the lipid hydrocarbon core proteins was calculated. ΔG transfer is the transfer of the energy of peripheral proteins from water to the lipid bilayer. The tilt angle—the angle between the membrane normal (Z axis) and protein axis. The last column presents models of analysed interactions. The red and blue discs represent the gram-negative’s outside cell membrane. The blue disc represents lipids directed to the periplasm.

### The digital assemblies of tail spike tip ends of analysed bacteriophages

The obtained results suggest that the small immunoglobulin-like proteins (grey models) and hypothetical hand-like proteins (green models) may have been functionally misannotated. Based on our analyses, we hypothesized that these proteins represent previously uncharacterized structural components of the tail spike receptor-binding system. To test this hypothesis, we performed in silico simulations of tail spike assembly using heteromeric modelling with AlphaFold2-Multimer.

**Figure 3:**
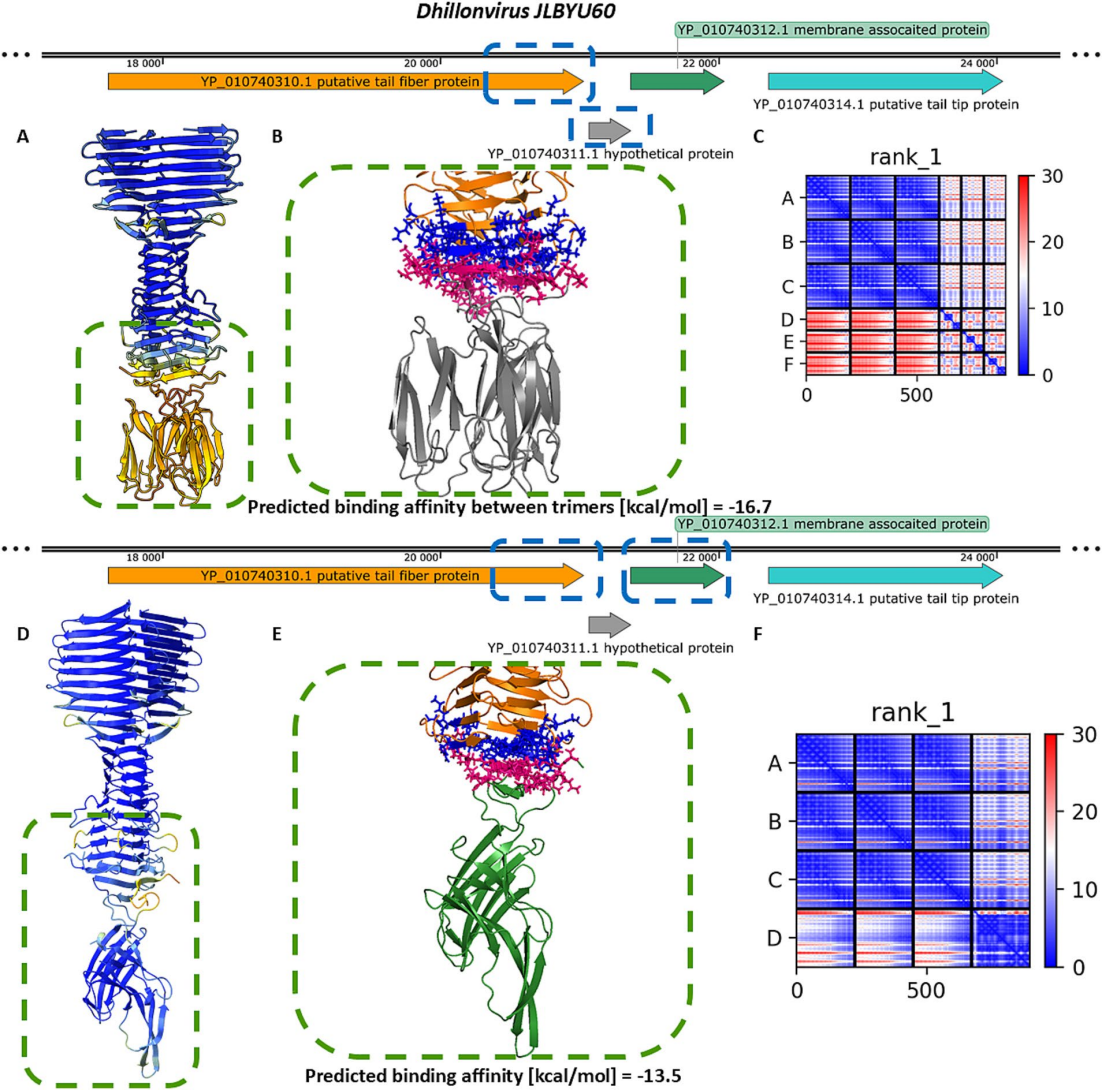
The analysis of modelling results of the interaction between the tail spike C-terminal domain in the native homo-trimer state and the downstream encoded proteins of *Dhillonvirus JLBYU60*. **(A,D)** The results of the modelling coloured by pLDDT. **(B**,**E)** The results of the analysis of the protein–protein interface with binding affinity between analysed proteins. **(C,F)** The matrix of PAE error score between predicted domains. The chains **A–C** (The tail spike C-terminal domain).

For *Dhillonvirus JLBYU60*, heteromeric modelling of the tail spike C-terminal homo-trimer in complex with the hypothetical protein YP_010740311.1 (also in a homo-trimeric form) resulted in a stable structure with a low Predicted Aligned Error (PAE) score at the interaction interface. The predicted binding affinity (ΔG) for this interaction suggested a thermodynamically favourable and potentially stable complex (Figures 3A–C).

Additional modelling of the tail spike C-terminal domain with monomeric YP_010740312.1 yielded a heteromeric structure with a higher predicted local distance difference test (pLDDT) score (Figure 3D) compared to the complex involving YP_010740311.1. Interface analysis indicated that the YP_010740312.1 monomer interacts with all three chains of the tail spike. Although the predicted binding affinity was slightly lower (Figure 3E), it was still sufficient to indicate a stable interaction. Furthermore, the PAE score for this complex was lower (Figure 3F), suggesting a more reliable structural model.

**Figure 4:**
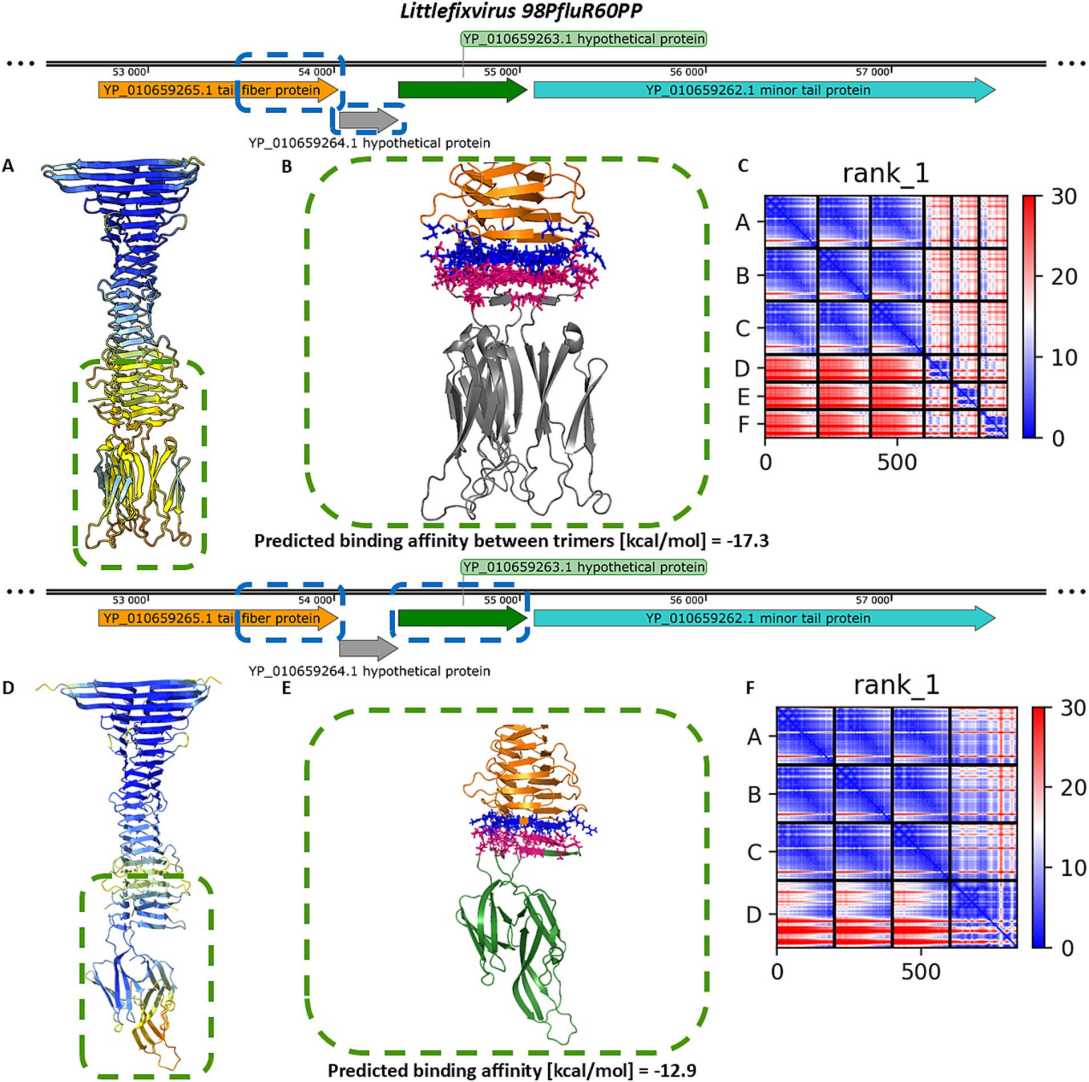
The analysis of modelling results of the interaction between the tail spike C-terminal domain in the native homo-trimer state and the downstream encoded proteins of *Littlefixvirus 98PfluR60PP*. **(A,D)** The results of the modelling coloured by pLDDT. **(B**,**E)** The results of the analysis of the protein–protein interface with binding affinity between analysed proteins. **(C,F)** The matrix of PAE error score between predicted domains. The chains **A–C** (The tail spike C-terminal domain).

Similar modelling of the *Littlefixvirus 98PfluR60PP* tail spike showed comparable results to those obtained for *Dhillonvirus JLBYU60* (Figure 4). The tail spike C-terminal domain was able to form stable complexes with both the homo-trimer of YP_010659264.1 and the monomer of YP_010659263.1. Notably, the interaction with YP_010659263.1 produced better pLDDT and PAE scores (Figures 4A,C,D,F) compared to YP_010659264.1. However, the predicted binding affinity was slightly higher for the homo-trimeric interaction (Figures 4B,E).

**Figure 5:**
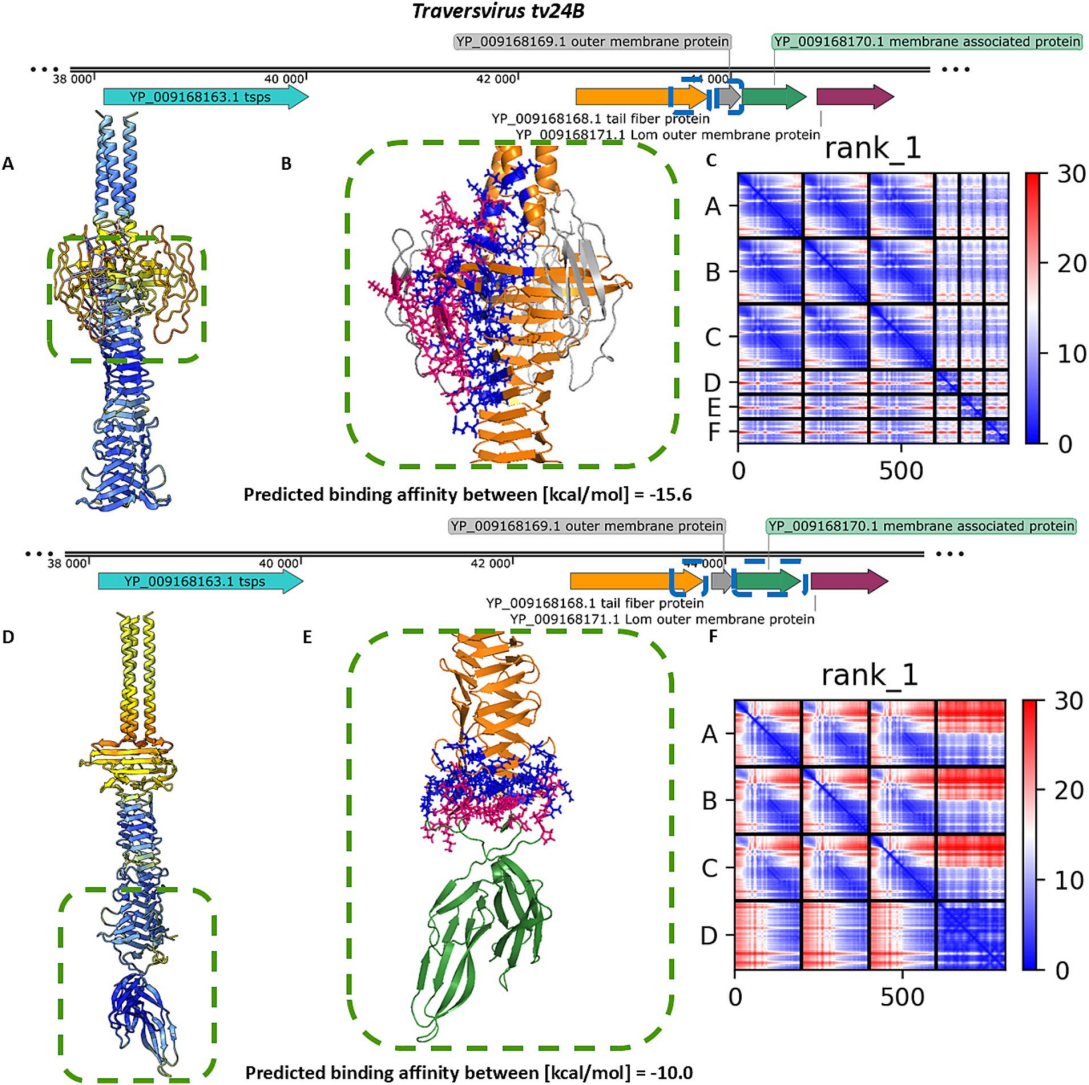
The analysis of results modelling results of the interaction between the tail spike C-terminal domain in the native homo-trimer state and the next coding proteins encoded by *Traversvirus tv24B*. **(A,D)** The results of the modelling coloured by pLDDT. **(B**,**E)** The results of the analysis of the protein–protein interface with binding affinity between analysed proteins. **(C,F)** The matrix of PAE error score between predicted domains. The chains **A–C** (The tail spike C-terminal domains).

For *Traversvirus tv24B*, heteromeric modelling of the interaction between the tail spike C-terminal domain and YP_009168169.1 did not yield a structure resembling the immunoglobulin-like trimeric complexes observed in other phages. Nonetheless, the model predicted a localized interaction at the base of the knife-like domain, with strong binding affinity and favorable PAE scores (Figures 5A–C). Modelling of the interaction with the hand-like protein YP_009168170.1 produced better structural confidence (higher pLDDT and lower PAE scores), although the predicted binding affinity was lower (Figures 5D–F).

**Figure 6:**
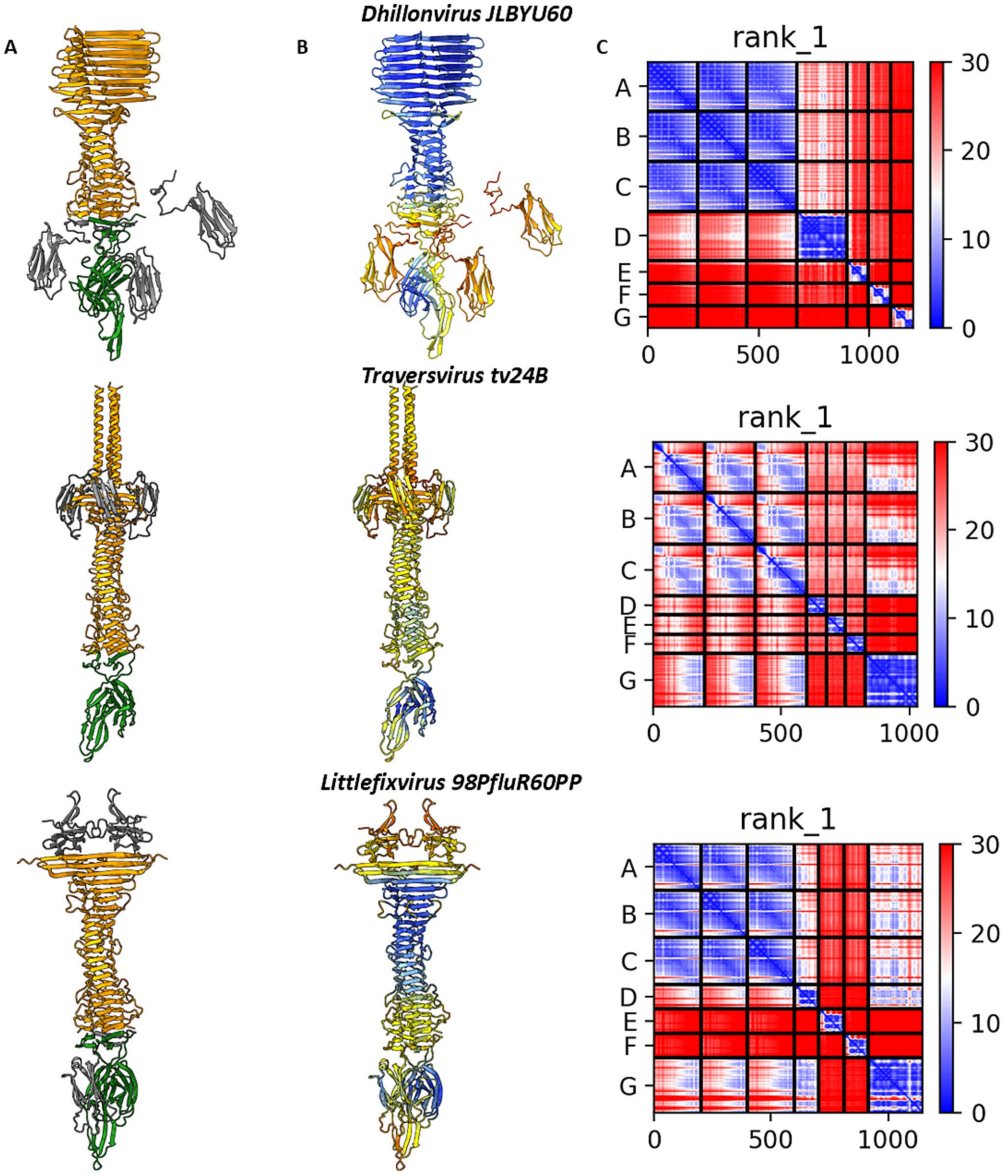
The results of digital assembly of bacteriophages’ tail spikes. **(A)** The cartoon models of the best results of heteromer modelling with coloured tail spike element: orange–tail spike homo-trimer C-terminal domain, grey—three copies of immunoglobulin-like hypothetical protein structurally similar to C-terminal domain of *lambda* gpJ, green—hypothetical protein annotated as membrane-associated protein. **(B)** The models’ per-residue confidence scores pLDDT. **(C)** Domain position confidence PAE. *Dhillonvirus JLBYU60* chains ID: tail spike C-terminal domain YP_010740310.1 (chains **A–C**), immunoglobulin-like protein YP_010740311.1 (chains **E–G**), membrane-associated protein YP_010740312.1 (chain **D**). *Traversvirus tv24B* chains ID: tail spike C-terminal domain YP_009168168.1 (chains **A–C**), immunoglobulin-like protein YP_009168169.1 (chains **D–F**), membrane-associated protein YP_009168170.1 (chain G). *Littlefixvirus 98PfluR60PP* chains ID: tail spike C-terminal domain YP_010659265.1 (chains **A–C**), immunoglobulin-like protein YP_010659264.1 (chains **D–F**), membrane-associated protein YP_010659263.1 (chain **G**).

Altogether, modelling the spatial arrangement of the tail spike and associated hypothetical proteins did not conclusively determine the precise interaction mode. To gain further insight, we modelled interactions involving all candidate proteins for each phage. Across all analysed cases, AlphaFold2-Multimer consistently favoured tail spike termination with hand-like proteins rather than immunoglobulin-like homo-trimers. Both pLDDT and PAE scores were more favourable for the hand-like protein complexes (Figure 6), supporting our hypothesis that these structures may represent preferred or more stable configurations.

### The comparison of the functional annotation

The newest bioinformatics tools for phage genome functional annotation are based on the PHROG database and use sequence or structure homology for protein function detection. Table 2 presents the comparison of functional annotation performed on the ‘newest’ phage proteins functions predictor implemented in the Sphea workflow. The ‘hand-like’ proteins from phages *Dhillonvirus JLBYU60* and *Traversvirus tv24B* matching to PHROG 425 ‘membrane associated protein’ and the same functional annotation are assigned by pharokka, phold and phynteny. In the case of YP_010659263.1 from *Littlefixvirus 98Pflur60PP* the PHROG was not detected, pharokka annotated protein as ‘hypothetical’, phold and phynteny predict function as membrane associated protein.

**Table 2:** The functional annotation of the ‘hand-like’ and the ‘Ig-like’ proteins.

Phage name	Genome ID	Protein ID	PHROG	PHROG name	Pharokka	Phold	Phynteny
The ‘hand-like’ protein							
Dhillonvirus JLBYU60	NC_073052.1	YP_010740312.1	425	Membrane associated protein	Membrane associated protein	Membrane associated protein	Membrane associated protein
Traversvirus tv24B	NC_027984.1	YP_009168170.1			Membrane associated protein	Membrane associated protein	Membrane associated protein
Littlefixvirus 98Pflur60pp	NC_070866.1	YP_010659263.1	ND	ND	Hypothetical protein	Membrane associated protein	Membrane associated protein
The ‘Ig-like’ small protein							
Traversvirus tv24B	NC_027984.1	YP_009168169.1	557	Outer membrane protein	Outer membrane protein	Outer membrane protein	Outer membrane protein
Dhillonvirus JLBYU60	NC_073052.1	YP_010740311.1	ND	ND	Outer membrane protein	Outer membrane protein	Outer membrane protein
Littlefixvirus 98Pflur60pp	NC_070866.1	YP_010659263.1			Hypothetical protein	Membrane associated protein	Membrane associated protein

The table consists of results analysis performed by PHROG sequence search, pharokka, phold and phynteny.

In the case of the ‘Ig-like” small protein performed analysis matching protein YP_009168169.1from *Traversvirus tv24B* to PHROG 557 ‘outer membrane protein’ and functional annotated by all softwares as outer membrane protein. In the case of YP_010740311.1 from *Dhillonvirus JLBYU60* and YP_010659263.1 from *Littlefixvirus 98Pflur60PP* matching frog were not detected, but YP_010740311.1 was functional annotated by all used software as ‘outer membrane protein’. In the case of YP_010659263.1 Pharokka did not predict protein function, but phold and phynteny annotated protein as membrane associated protein.

### The prediction of the interaction between bacteriophage proteins and receptors on the surface of the bacteria

**Figure 7:**
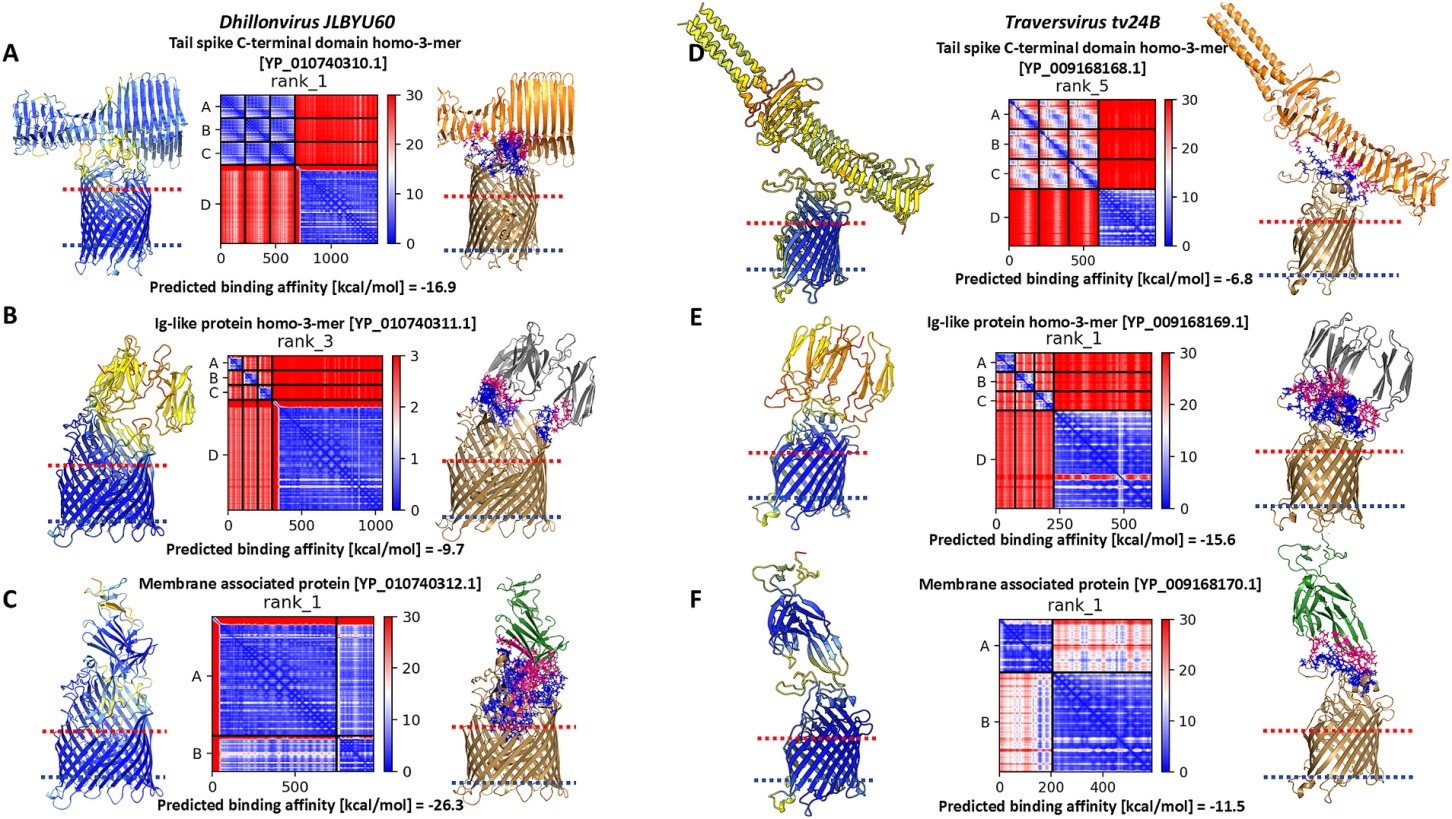
The results of modelling of interaction between *Dhillonvirus JLBYU60* proteins and FhuA, and the results of modelling of interaction between *Traversvirus tv24B* proteins and transmembrane domain of BamA. For each layout presented pLDDT score, PAE error matrix score and interaction interface. **(A)** The best model of interaction between tail spike C-terminal domain and FhuA. **(B)** The best model of interaction between YP_010740311.1 and FhuA which interact with FhuA flexible loops presented outside the cell. **(C)** The best model of interaction between the hand-like protein (YP_010740312.1) and FhuA. **(D)** The best model of interaction between tail spike C-terminal domain and BamA transmembrane domain. **(E)** The best model of interaction between YP_009168169.1 and BamA. **(F)** The best model of interaction between the hand-like protein (YP_009168170.1) and BamA.

For *Dhillonvirus JLBYU60*, experimental confirmation of the main receptor was provided by Jessica *M. Lewis* and colleagues (Lewis et al., 2023), who identified the ferrichrome outer membrane transporter (FhuA) as the main receptor for this phage. In the present study, we performed structural modelling of interactions between candidate phage proteins and the FhuA receptor, as shown in Figures 7A–C.

Heteromeric modelling of the tail spike C-terminal domain revealed a potential interaction interface between the knife-like domain and the flexible extracellular loops of FhuA. The predicted binding affinity for this interaction was −16.9 kcal/mol. However, the modelled interface configuration suggests the parallel position of the tail spike to the bacterial cell surface, potentially limiting accessibility. The associated PAE matrix showed a high error score at the interaction site, indicating lower confidence in the predicted interface (Figure 7A).

Modelling the interaction between the homo-trimeric YP_010740311.1 and FhuA resulted in the lowest pLDDT score, the highest PAE error, and the weakest predicted binding affinity among the tested candidates (Figure 7B).

In contrast, interaction modelling between the hand-like protein YP_010740312.1 and FhuA showed a markedly higher pLDDT score and a low PAE error score across the binding interface. The predicted binding affinity indicated a strong and thermodynamically favorable interaction between YP_010740312.1 and FhuA (Figure 7C).

For *Traversvirus tv24B*, the receptor has been experimentally identified as the β-barrel transmembrane domain of BamA (Smith et al., 2007). Our modeling of the interaction between the tail spike C-terminal domain and BamA β-barrel domain resulted in the lowest pLDDT score, highest PAE error, and weakest predicted binding affinity of the analysed complexes (Figure 7D). Similar to *Dhillonvirus*, the interaction geometry positioned the receptor-binding system nearly parallel to the bacterial surface.

In contrast, modelling of the homo-trimeric YP_009168169.1 in complex with the C-terminal domain of BamA produced a low pLDDT score and high PAE error, but a high predicted binding affinity—likely due to multiple contact points across the interface (Figure 7E). The most favorable interaction was observed for YP_009168170.1: this model achieved the highest pLDDT score and the lowest PAE error at the binding interface. Although its predicted binding affinity was lower than that of the Ig-like trimer model, it remained within a thermodynamically plausible range (Figure 7F).

### The simulation of stability of predicted tail spike tip ends by molecular dynamics simulation (MDS) in 1 μs time scale

To evaluate the stability of the predicted protein complexes and determine whether they represent realistic assemblies rather than artifacts of computational modelling, molecular dynamics (MD) simulations were performed. The simulations were conducted in an aqueous environment at 300 K over a 1 μs timescale.

**Figure 8:**
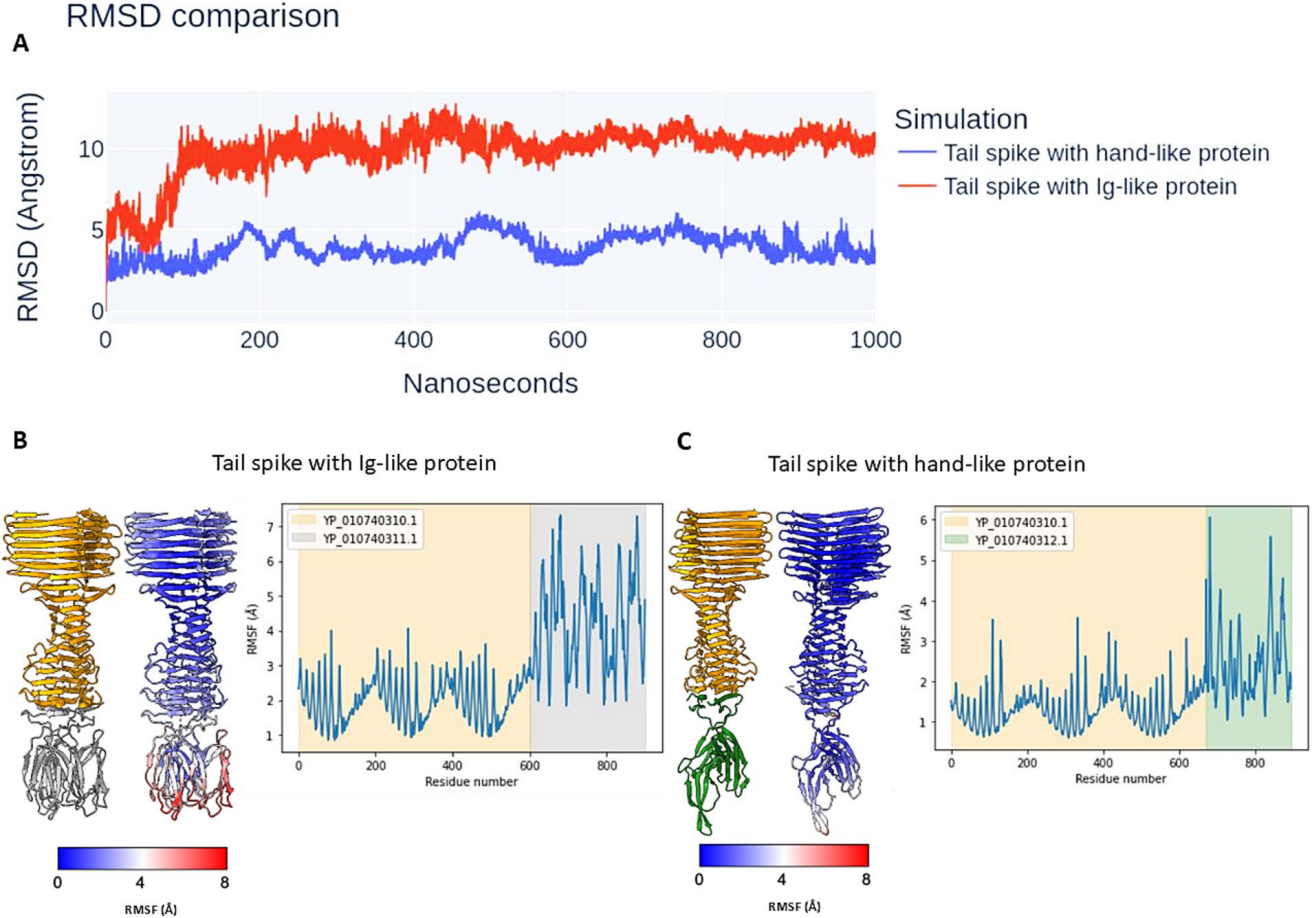
The results of the MDS of analysed protein complexes. **(A)** The RMSD comparison. **(B**,**C)** The atomic RMSF during 1 μs simulation. The complexes are coloured by subunits (left models) and RMSF attribute (right models). The graphs are coloured by elements in models.

Root-mean-square deviation (RMSD) analysis (Figure 8A) indicated that the complex formed between the tail spike C-terminal domain and the hand-like protein remained more stable throughout the simulation compared to the complex with the Ig-like protein. The latter underwent significant structural rearrangements within the first 100 ns of the simulation, with deviations exceeding 10 Å, before reaching a more stabilized state. In contrast, the RMSD of the hand-like protein complex remained around 5 Å throughout the full 1 μs simulation.

Root-mean-square fluctuation (RMSF) analysis further supported these findings. The tail spike C-terminal domain complexed with the Ig-like protein exhibited greater overall flexibility, particularly in the peripheral regions of the Ig-like domains, which showed fluctuations exceeding 7 Å (Figure 8B). Conversely, the complex with the hand-like protein displayed significantly lower RMSF values, indicating greater structural rigidity. The two regions of highest flexibility in this complex (RMSF > 5 Å) corresponded to external loop regions of the hand-like protein (Figure 8C).

A video of the MD simulation for both structural layouts is available in Supplementary material 2.

### The SDS-PAGE analysis of *Littlefixvirus 98PfluR60PP* virion proteins

Protein modelling results suggest that the tail spikes of the analysed phages may be terminated by an additional protein exhibiting high affinity for receptors on the bacterial cell surface. However, these in silico predictions require experimental validation.

**Figure 9:**
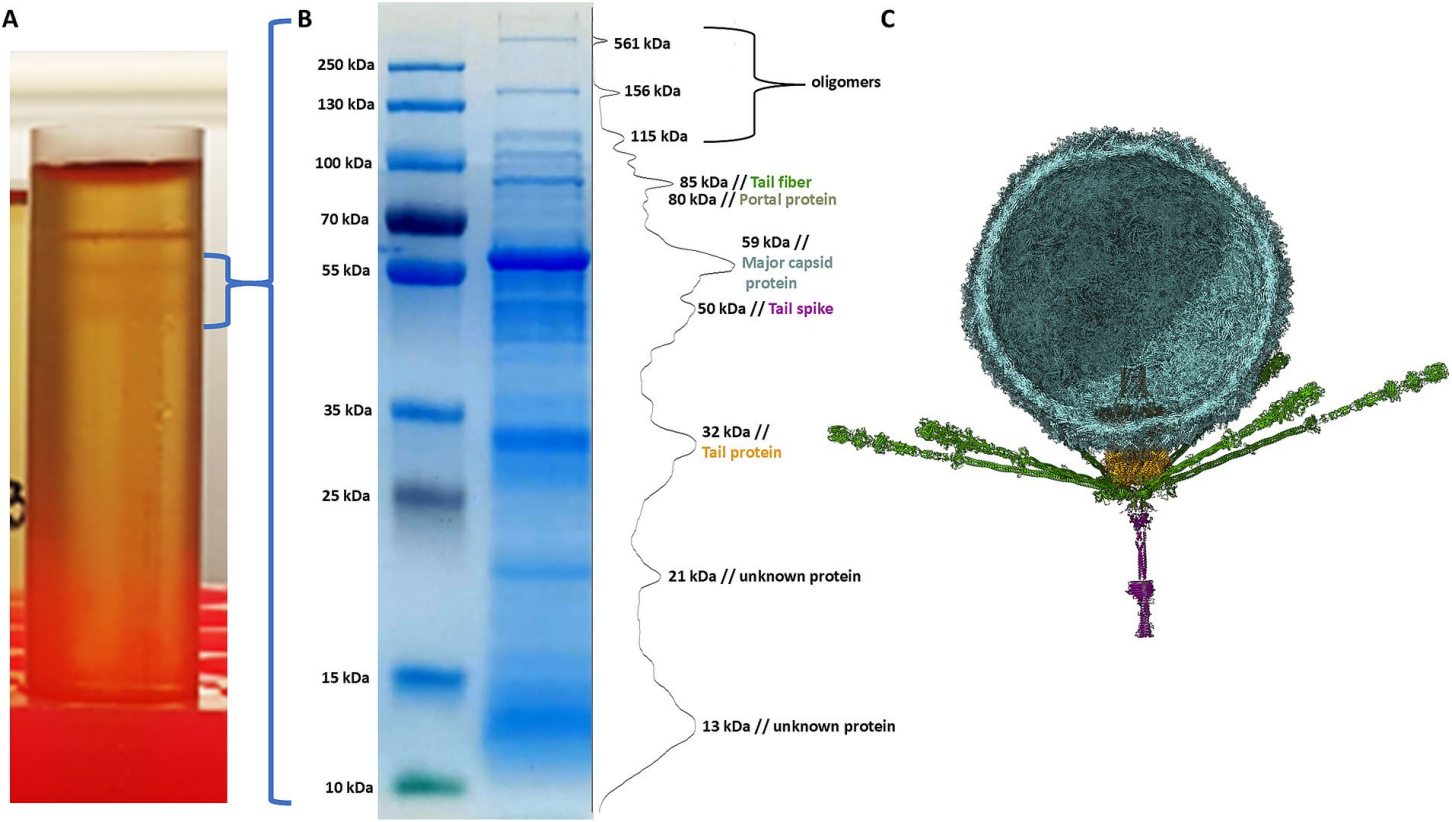
The results of analysis *Littlefixvirus 98PfluR60PP virion* proteins. **(A)** The phage fraction after ultracentrifugation in CsCl gradient. **(B)** The SDS-PAGE results with bands intensity graph. **(C)** The bacteriophage digital reconstruction (unpublished data) showing the position and oligomeric state of the detected proteins.

The phage sample after ultracentrifugation is presented in Figure 9A, the lower grey bands were collected and analysed. To assess the correlation between theoretically identified structural proteins and those isolated from virions, SDS-PAGE analysis was performed for *Littlefixvirus 98PfluR60PP* (Figure 9B). Figure 9C presents the digital reconstruction of the analysed virions. This phage was originally described by Kazimierczak and colleagues as an effective and safe agent against pathogenic, antibiotic-resistant Pseudomonas spp. in the study (Kazimierczak et al., 2019). The transmission electron microscopy picture of this bacteriophage is available in this thesis.

The SDS-PAGE results were compared with the theoretical molecular masses of structural proteins predicted through functional annotation, allowing for the tentative identification of the observed bands (Table 3). A prominent band at approximately 21 kDa corresponded well with the hypothetical protein YP_010659263.1—identified in this study as a candidate hand-like protein potentially acting as a missing receptor-binding element.

**Table 3:** The results of a molecular weight analysis of *Littlefixvirus 98PfluR60PP* proteins by SDS-PAGE.

Protein name	GeneBank ID	Theoretical MW [kDa]	SDS-PAGE [kDa]	MW Diff
Tail fiber	YP_010659262.1	85	85	0%
Portal protein	YP_010659288.1	80	80	0%
Major capsid protein	YP_010659285.1	51	59	15%
Tail spike	YP_010659265.1	46	50	8%
Tail protein	YP_010659292.1	28	32	15%
Unknown protein	YP_010659263.1	25	21	−16%
Unknown protein	?	Unknown	13	-

Additionally, a diffused band was observed around 13 kDa, which may correspond to the hypothetical Ig-like protein or alternatively to a minor capsid or scaffolding protein. The intensity of this band suggests a relatively high abundance of the protein in the virion, supporting the possibility that it plays a structural role, potentially as a minor capsid component or internal scaffold.

## Discussion

Bacteriophage receptor-binding proteins (RBPs) are among the most specialised and evolutionarily dynamic components of phage virions, enabling the specific recognition of host cells (Supplementary material 1; Supplementary Figures 1, 2). These proteins have been shaped by the ongoing evolutionary arms race between phages and their bacterial hosts. Accurate functional annotation of such proteins is critical, particularly in the current era of machine learning–based prediction tools, which depend heavily on the quality of input data. The results of the comparison of the functional annotations show that the ‘newest’ annotators based on PHROGS database did not detect protein functions, or these proteins were annotated these proteins as membrane proteins. These studies show bioinformatic evidence that these proteins have a significantly lower membrane affinity compared to affinity to the central phage tail spike, which suggests that they are the virion’s structural elements.

In this study, we performed a comprehensive bioinformatic investigation of RBPs that interact with outer membrane proteins in three distantly related bacteriophages: *Dhillonvirus JLBYU60*, *Traversvirus tv24B* and *Littlefixvirus 98PfluR60PP*. Despite their phylogenetic distance, these phages display a similar tail spike architecture. Our modelling and simulation analyses suggest that the tail spike proteins of these phages have relatively low binding affinity for their receptors and, when binding, are likely to adopt an orientation nearly parallel to the bacterial cell surface—resulting in potential steric clashes with surface elements such as LPS or other membrane proteins (Supplementary material 1; Supplementary Figure 4).

Molecular modelling and dynamics simulations indicated that tail spikes may form stable complexes with additional proteins—either homo-trimeric immunoglobulin-like domains or the so-called hand-like proteins. Among these, the hand-like protein complexes demonstrated slightly greater stability over 1 μs simulations. Furthermore, the predicted binding affinities of these complexes were comparable to those of experimentally characterized phage tail fiber and tail spike complexes (Figures 3–5; Supplementary Figure 3).

Recent structural studies of *Lambdavirus lambda* by Ge and Wang (2024) highlighted the importance of homo-trimeric immunoglobulin-like domains for receptor recognition, specifically for binding to the LamB trimer. In contrast, the *Tequintavirus T5* tail spike—although also possessing C3 symmetry—requires an additional RBP (pb5) for receptor binding (Degroux et al., 2023). Based on these findings, we propose two hypotheses:

First one: if both analyzed hypothetical proteins (Ig-like and hand-like) are expressed during phage assembly, the phage population may be divided into subpopulations with tail spikes terminated either by Ig-like homo-trimers (targeting one receptor type) or hand-like proteins (targeting another). Such a mechanism could broaden host range and serve as a strategy to overcome phage resistance via receptor mutation.

Alternatively, only one of these proteins—likely the hand-like protein—is expressed and serves as the sole receptor-binding module, forming a more stable complex with the tail spike and showing high binding affinity to the FhuA receptor, as observed in *Dhillonvirus JLBYU60*.

Notably, we identified structurally similar hand-like proteins in other phage genera, including *Kuravirus*, which was previously characterized by Šiborová et al. (2022). Although this team resolved a nearly complete virion structure, low electron density in the tail fiber regions precluded structural resolution of these components. No additional proteins were identified at the tail spike end, but this may reflect structural averaging during Cryo-EM reconstruction. We hypothesize that key tail spike details may be lost during Cryo-EM averaging of single-particle images (Supplementary material 1; Supplementary Figure 5).

A similar case was observed in *Pseudomonas* phage DEV (species *Litunavirus Ab09*), as described by Ravi K. Lokareddy et al. (2024). While major virion components were reconstructed, the low resolution of the tail fiber and tail spike regions limited structural interpretation to model-based predictions.

**Figure 10:**
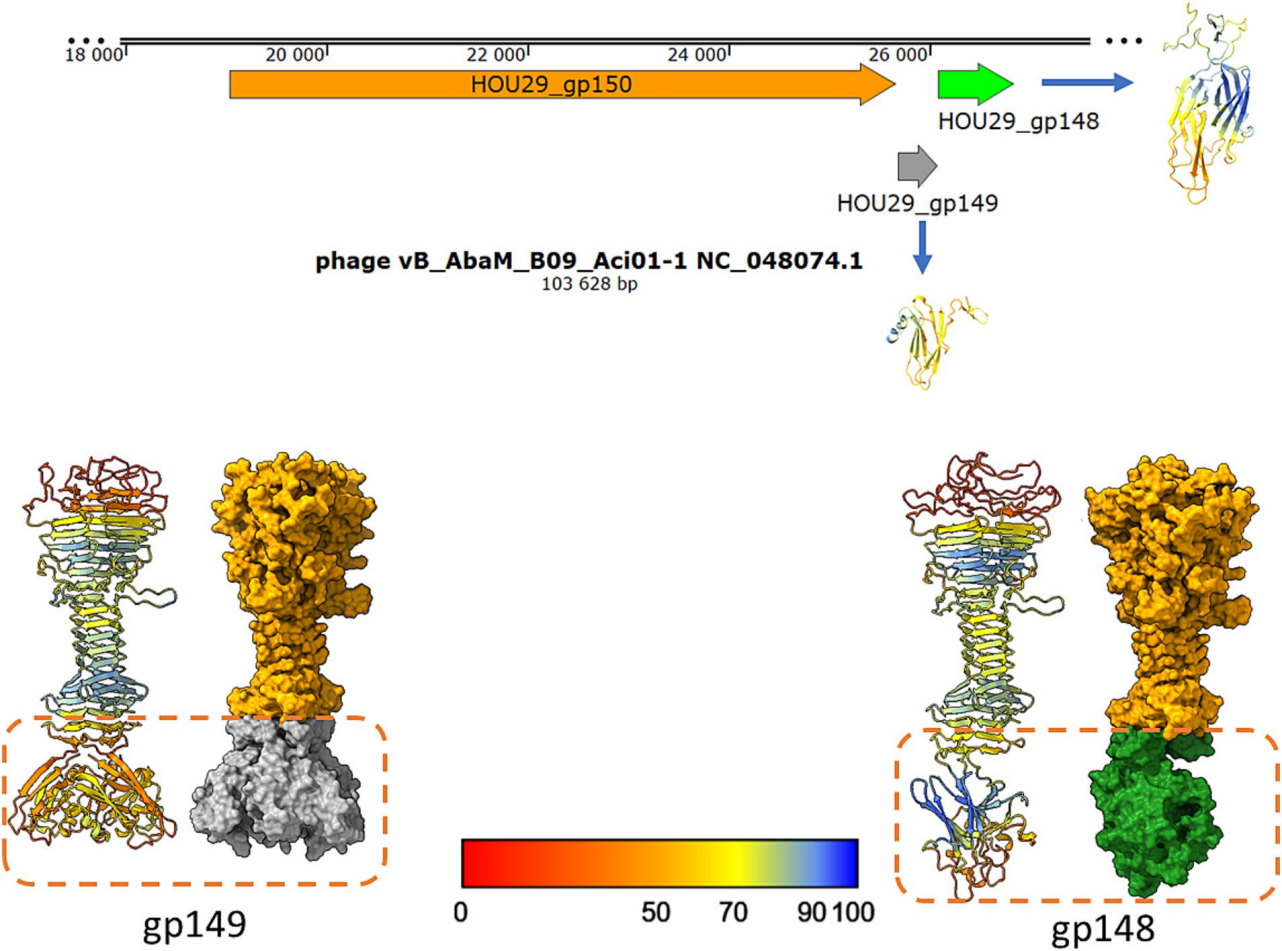
The model of postulated *Saclayvirus Aci01-1* long tail spike end, ended by small Ig-like trimer or hand-like protein similar to phages analysed in this studies. The shape of the suggested complexes are similar to electron density under globular domain 7 on the Figure 1A presented by Pourcel et al. (2023) in *Archives of Virology* (Pourcel et al., 2023).

Pourcel et al. (2023) published a brief report describing *Saclayvirus Aci01-1*, a bacteriophage infecting *Acinetobacter baumannii*, which features an unusually long and complex tail fiber (referred to as a “tail spike” in our study). High-resolution electron micrographs in that work revealed multiple globular sugar-binding domains (Pourcel et al., 2023). Comparison between their experimental micrographs and AlphaFold2-generated models revealed discrepancies at the distal end of the tail spike. Additional globular domains visible in the micrographs were not predicted by AlphaFold2 (Figure 1 vs. Figure 3, Pourcel et al., 2023, *Archives of Virology*). Our reanalysis of the *Aci01-1* genome identified two ORFs downstream of the tail spike gene encoding small immunoglobulin-like and hand-like proteins structurally similar to those investigated in this study. Structural modelling of putative heteromeric complexes incorporating these proteins revealed shapes consistent with the unassigned electron density at the spike terminus (Figure 10 of this study and Figure 1, Pourcel et al., 2023).

We are aware that our analysis was performed on a small data set, and to confirm the existence of the analysed proteins, it would be necessary to conduct an in-depth analysis of a larger number of phages possessing a non-contractile tail with a missing receptor binding domain. However, at present, we are not able to conduct broader studies and laboratory verification of the performed simulations. We acknowledge that bioinformatic analyses alone are insufficient to definitively confirm the proposed hypotheses regarding tail spike architecture in these bacteriophages. These hypotheses must be validated through structural and experimental approaches. The main objective of this study is therefore to emphasize the need for a more detailed exploration of virion components involved in receptor recognition—specifically, tail spike-associated proteins. We propose that a reanalysis of the electron microscopy datasets cited here may help support or refute the structural predictions presented in this work.

## Conclusion

The results of this study support the hypothesis that current functional annotation methods may be insufficient to accurately identify receptor-binding domains within phage tail spikes. While tail spikes are typically expected to terminate in immunoglobulin-like (Ig-like) domains—as exemplified by *Lambdavirus lambda*—the phages analysed in this study lack such domains in their annotated tail spike proteins.

Our bioinformatic analyses suggest that the tail spikes of these phages may be connected with additional partner proteins to mediate receptor recognition. Two candidate proteins were identified: (1) a homo-trimer of a small Ig-like protein (~11 kDa), encoded by the first ORF downstream of the tail spike gene, and (2) a previously uncharacterized “hand-like” protein (~21 kDa), encoded by the second downstream ORF.

Structural modelling and molecular dynamics simulations demonstrated that both protein complexes (tail spike + Ig-like or hand-like proteins) remain stable over the course of 1 μs simulations, though they exhibit differing degrees of flexibility. Binding affinity analyses revealed that, depending on the phage system, either the hand-like protein or the Ig-like trimer displayed higher predicted affinity for the receptor.

While our results provide supportive evidence for the hypothesis that tail spikes lacking Ig-like domains may require accessory proteins for receptor binding, definitive confirmation will require experimental validation. Further laboratory studies are essential to verify the structural roles of these candidate proteins and to fully elucidate the architecture of receptor-binding systems in bacteriophages.

## Data Availability

Publicly available datasets were analyzed in this study. This data can be found here: https://www.ncbi.nlm.nih.gov, accession numbers NC_005859.1; NC_001416.1; NC_073052.1; NC_027984.1; NC_070866.1.

## Footnotes

^1^ https://swissmodel.expasy.org/

^2^ https://opm.phar.umich.edu/ppm_server

^3^ https://github.com/gbouras13/phold

^4^ https://rascar.science.uu.nl/prodigy/

## Supplementary material

The Supplementary material for this article can be found online at: https://www.frontiersin.org/articles/10.3389/fmicb.2025.1625765/full#supplementary-material and Supplementary material 2 at https://zenodo.org/records/15371830
